# Prevalence of alcohol use in late pregnancy

**DOI:** 10.1038/s41390-019-0731-y

**Published:** 2020-01-03

**Authors:** Amna Umer, Christa Lilly, Candice Hamilton, Aileen Baldwin, Janine Breyel, Amy Tolliver, Christina Mullins, Collin John, Stefan Maxwell

**Affiliations:** 1 Department of Pediatrics, School of Medicine, West Virginia University, Morgantown, WV 26506 USA; 2 Department of Biostatistics, School of Public Health, West Virginia University, Morgantown, WV 26506 USA; 3 United States Drug Testing Laboratories, Inc, Des Plaines, IL USA; 4 West Virginia Perinatal Partnership, Charleston, WV 25301 USA; 5 Office of Maternal, Child and Family Health, West Virginia Department of Health and Human Resources, Charleston, WV USA; 6 Department of Pediatrics, WV School of Medicine (Charleston division) PEDIATRIX Medical Group, Women & Children’s Hospital, Charleston, WV USA

## Abstract

**Background:**

Prenatal alcohol exposure (PAE) can result in detrimental developmental complications. The objective of this study was to estimate the most recent PAE prevalence data for the state of West Virginia (WV) and associated factors.

**Method:**

In all, 1830 newborn residual dried blood spots (DBS) in the WV Newborn Screening Repository were analyzed for phosphatidylethanol (PETH). Data were matched with Project WATCH data (94% match, *N* = 1729).

**Results:**

The prevalence of late pregnancy PAE was 8.10% (95%CI: 6.81, 9.38) for all births, 7.61% (95%CI: 6.26, 8.97) for WV residents only, and ranged from 2.27 to 17.11% by region. The significant factors associated with PAE included smoking (OR: 2.03, 95% CI: 1.40, 2.94), preterm births (OR: 1.88; 95% CI: 1.23, 2.89), birth weight of ≤2000 g vs. >3000 g (OR: 2.62, 95%CI: 1.19, 5.79), no exclusive breastfeeding intention (OR: 1.45, 95% CI: 1.02, 2.04), and not exclusively breastfeeding before discharge (OR: 1.61; 95% CI: 1.09, 2.38).

**Conclusion:**

The prevalence of PAE is higher than previously shown for the state. Accurate and timely estimates are vital to inform public health workers, policymakers, researchers, and clinicians to develop and promote effective prevention strategies to lower PAE prevalence and provide targeted interventions and treatment services for infants affected by PAE.

## Introduction

Maternal alcohol use in pregnancy negatively impacts several organ systems of the developing embryo and fetus.^1^ The severity of the outcomes varies by dose, duration, and the developmental stage of the embryo.^1^ There is a broad range of deficits associated with prenatal alcohol exposure (PAE) referred to as fetal alcohol spectrum disorders (FASD).^1^ FASD includes the fetal alcohol syndrome (FAS), which is the most severe and specific outcome associated with high-dose ethanol exposure in the first trimester during organogenesis. This includes but is not limited to growth deficiency, developmental delay, craniofacial malformations, and intellectual disabilities as described in 1973 by Smith and Jones.^2–4^ In the third trimester, after the phenotype has been established and the critical period of organogenesis is mainly complete, there is a period of rapid brain growth. Alcohol exposure during this period is associated with a range of developmental problems that may not be overtly expressed as morphological deformities. These include developmental problems, such as difficulties with visual-spatial learning, language, executive functioning, attention and focusing, memory skills, auditory comprehension, reaction time, and organizing or sequencing tasks.^5^ A recent US study among first-grade children [mean age 6.7 years (SD, 0.41)] in four US communities estimated the prevalence of FASD ranging from 1.1 to 5.0%.^6^ As PAE can have detrimental consequences at various gestational stages, there is no safe amount of alcohol intake throughout pregnancy,^5^ and complete abstinence is recommended.^7^ Though the outcomes and guidelines for alcohol use in pregnancy are well-established, the most recent National Survey on Drug Use and Health (NSDUH) self-reported survey for the years 2016–2017 showed a high prevalence of PAE in the United States (US).^8^ Nearly 11.5% of pregnant women drank any amount of alcohol in the past 30 days and the estimated incidence of prenatal binge drinking (defined as ≥4 or more alcoholic drinks on the same occasion on at least 1 day in the past month) was 5.2%.^8^

West Virginia (WV) is a primarily rural Appalachian state in the US and has high rates of chronic diseases, substance use disorder, and smoking during pregnancy relative to the rest of the nation. Based on the Pregnancy Risk Assessment Monitoring System (PRAMS) 2015 survey, 2.7% of women in WV reported drinking alcohol during their last 3 months of pregnancy.^9^ However, PRAMS is a self-reported telephone survey, and this method has shown to underestimate the prevalence of alcohol consumption by more than one third.^10^ Currently, there are no standard laboratory tests that can definitively detect alcohol use in pregnancy due to its rapid metabolism; thus, testing maternal blood, breath, or urine is only useful for alcohol exposure within the previous 24 h.^11^

Phosphatidylethanol (PETH) is a direct metabolite formed from alcohol (ethanol) consumption, manifesting as a phospholipid incorporated into the membranes of red blood cells.^12^ PETH from dried blood spots (DBS) has been shown to be a valuable test with a high degree of sensitivity for assessing PAE at birth.^12–15^ PETH has a long half-life of 3–5 days and a positive PETH value is indicative of late pregnancy PAE (2–4 weeks).^12–16^ Although there is a positive linear correlation between degree of alcohol consumption and PETH concentrations, there have been no established PETH cutoffs that differentiate various levels of alcohol consumption (low vs. moderate vs. heavy) during pregnancy.^17^ In 2009, an umbilical cord tissue study was conducted at eight hospitals in WV to estimate the rate of intrauterine substance exposure (IUSE) in the state. The study tested the tissue for various drug metabolites including PETH for late pregnancy PAE. The results showed that the prevalence of late pregnancy PAE across the eight hospitals was 5.1%.^18^ A recent study in Texas using the newborn residual DBS found 8.4% of the sample was positive for PETH (>20 ng/ml).^13^

Due to screening and diagnostic complexities for alcohol use in pregnancy, there are limited data on the statewide prevalence of PAE for WV. Self-reported surveillance systems such as PRAMS certainly underestimate the true prevalence rate of prenatal alcohol use, and the WV umbilical cord tissue study was conducted a decade ago from a limited number of hospitals. The objective of this study was to estimate the most recent prevalence data on late pregnancy PAE in the state of WV by screening newborn residual DBS. This method has shown to be sensitive and cost-effective when estimating prevalence of PAE in late pregnancy at a population-level.^13,19^ The prevalence data were compared to other estimated prevalence data for the state. The secondary aim of the study was to examine maternal and infant sociodemographic and health-related characteristics associated with PAE. There is increasing scientific evidence that up to 5% of children born in the US each year will demonstrate developmental disabilities related to FASD during early childhood. Thus, assessing the extent and nature of PAE as one of the most preventable cause of birth defects can have significant policy implications for the health and wellbeing of the newborns in the state of WV.

## Methods

### Data collection

#### Dried blood spot specimens

As part of the Newborn Screening Program, DBS cards are collected from all newborns from all birthing centers in WV and mailed to the WV Office of Laboratory Services (OLS) within 24 h after collection. The details of the data collection process are provided elsewhere.^20^

#### Study sample

To calculate the sample size needed for this study, we powered the study both for the precision of the prevalence estimate for the state of WV and for testing the prevalence rate relative to the other methods previously used (self-report and umbilical cord). These sample size estimates between 1105 and 1825 for detecting 8−5% prevalence meet or exceed the sample size necessary for testing a noninferiority test (i.e., showing "as good as or better") assessing the proportion of populace testing positive for alcohol use, using the new method as compared to umbilical cord. This study was approved by the West Virginia University IRB (protocol, #1712902480). The final study sample included randomly selected 610 DBS cards from each of the months of November (2017), December (2017), and January (2018) for a total of 1830 DBS from the state newborn screening repository of WV OLS. The newborn DBS cards were transferred to the Project WATCH office (aka West Virginia Birth Score) at West Virginia University. The Birth Score team provided a unique identifier for each DBS card that was separated at the dotted line from the newborn screening card that contained the newborn’s identifying information. The deidentifiable portion of the card containing the blood spots was sent to the United States Drug Testing Laboratories, Inc. (USDTL) for PETH analysis.

#### Project WATCH

Project WATCH is a WV statewide mandate since 1998 (House Bill 2388). The project collects surveillance data on every infant born in all WV birthing hospitals/facilities to identify infants who are at a higher risk of infant mortality the first year of life, in order to initiate close follow-ups. More information about this project can be found elsewhere.^21^ USDTL sent the PETH-DBS lab results to the Project WATCH office in mid-to-late 2018. The USDTL file was matched with the Project WATCH file using the infant’s last name and birth date. This matched 1709 records (92.98%). The remaining data were hand matched for another 20 infants, which made a total of 1729 matched out of the 1830 cases (94.17%).

### Measures

#### PETH analysis

PETH is a long-term biomarker of alcohol ingestion, which can be detected and measured in the DBS,^13^ and indicates PAE in the month prior to birth.^12^ The DBS specimens were analyzed at USDTL using previously published methods.^22^ The limit of detection (LOD) was 2 ng/ml; the limit of quantitation (LOQ) was 8 ng/ml and the assay was linear up to 200 ng/ml. For this study, given no amount of alcohol has been established as safe during pregnancy, a positive PETH result was defined as the LOQ of a reading of ≥ 8 ng/ml or 0.08 g in 100 ml. The cutoff was based on the works of Jones et al.^22^ and Baldwin et al.^12^ on alcohol detection in the DBS by USDTL. Other researchers have also used PETH cutoff at ≥ 8 ng/ml to indicate a positive and moderate to heavy alcohol use in the last month of pregnancy.^14,16,23^ Using this analytical cutoff, we can capture all data for any exposure with a high degree of sensitivity and specificity.

#### Project WATCH data

Maternal factors from Project WATCH included: (1) maternal age (<19 and ≥19), race (white and others), education (≤10 grades and ≥11 grades), health insurance status (Medicaid and Private), smoking during pregnancy (yes/no), feeding intention (breast only, bottle or both), gestational age (preterm and term), and number of previous pregnancies (0 and ≥1). The infant variables included birth weight (≤2000, 2001–2500, 2501–3000, and >3000 g), IUSE (yes/no), neonatal abstinence syndrome (NAS) (yes/no), infant exclusively breastfed before hospital discharge (yes/no), and NICU admission (yes/no).

#### Region

WV consists of 55 counties, which are divided into six Substance Abuse and Mental Health Services Administration (SAMHSA) regions. SAMHSA works with the state substance abuse and mental health agency to define substate regions that meet the state needs and reporting requirements while ensuring the sample size for the NSDUH is large enough to provide estimates with adequate precision.^24^ These substate regions were created to understand the geographic variability of substance use data within each state in order to provide vital information for planning, reporting, program development, prevention and intervention efforts, and allocation of funds to areas in need for services.^24^ The substance use data are mostly analyzed using these regions due to the availability of resources and services in the counties clustered together in one region. This substate regional variable was created from the Project WATCH data that contains county-level information of the mother at the time of delivery.

### Analysis

Analysis for this project included the proportion with high PETH (≥8 ng/ml) for the total sample of all births in the state of WV, WV residents’ only, and by WV SAMHSA regions, with alpha set to 0.05. Exact confidence intervals (CI) are also reported. We also present the sociodemographic characteristics of the entire sample and also by six SAMSHA regions. Logistic regression analysis was conducted to examine the bivariate association between late pregnancy PAE and several maternal and infant characteristics and presented as odds ratio (OR) along with 95% CIs.

The prevalence rates for the state were also tested against known values from other studies using noninferiority testing, with the research hypothesis that the estimate would be higher or the same as other methodologies that may underestimate the true prevalence rates.^25^ Noninferiority testing is a statistical methodology where one tries to demonstrate similarity rather than difference to an established result.^25^ Specifically, noninferiority testing tests the research hypothesis that the new method or therapy is equivalent or superior (i.e., “as good as or better”, or not inferior) to the current one, and the null hypothesis that it is inferior.^26^ Thus, the series of noninferiority tests were run by the binomial test of one proportion against the primary hypothesis value based on the WV 2009 umbilical cord study (5.1%)^18^ as well as proportions found by self-reported PRAMS state data (WV data; 3.7%)^9^ and by a similar study in a different state (8.4% with >20 ng/ml, 24.7% with ≥ 8 ng/ml),^13^ with a margin of 3% and alpha set to 0.05. Results within the lower confidence interval margin of 3% and larger would establish equivalence or superiority to the previously established prevalence rates (i.e., the method would be as “good as or better than” the methods which resulted in the previously reported prevalence). Anything below the 3% lower confidence interval margin would be considered “inferior” or within the null hypothesis.

Four post-hoc analyses were performed to understand and explain the study findings. These include (1) logistic regression analyses to examine the association of late pregnancy PAE and birth weight in data restricted to full-term births only (*n* = 1498, 87%, (2) logistic regression analyses to examine the association of late pregnancy PAE and term birth stratified by infant sex, (3) calculate the proportion of alcohol use data captured within the substance use data collected by Project WATCH and (4) examine the prevalence of PAE using a higher cutoff of >20 ng/ml, as conducted by Bakhireva et al. for comparison purposes.^13^

## Results

The final sample size was 1729. The prevalence of late pregnancy PAE (PETH ≥8 ng/ml) was 140 cases or 8.10% (95% CI: 6.81, 9.38) in the entire sample that included out-of-state residents as well. The positive PETH concentrations ranged from 8 to 346 ng/ml with a mean positive PETH concentration of 20.7 ng/ml (SD = 34.6). Nearly 15% of infants were born to mothers who lived in the surrounding states of Kentucky, Maryland, Ohio, Pennsylvania, and Virginia but gave birth in the state of WV.

**Fig. 1:**
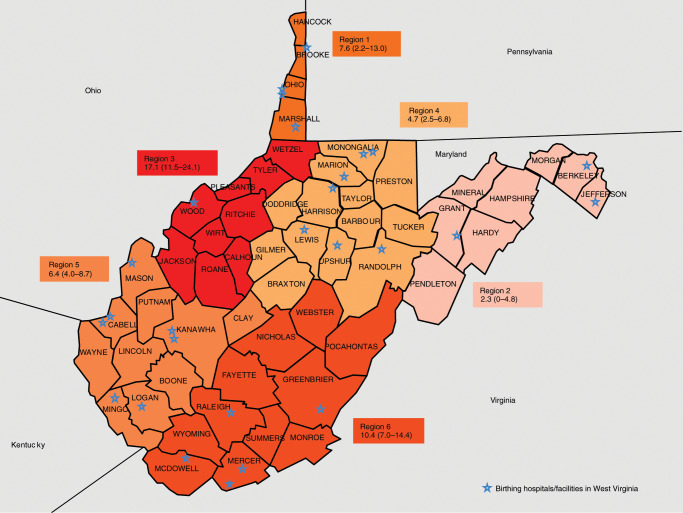
Prevalence and 95% confidence limits of late prenatal alcohol use by SAMHSA substate regions for West Virginia residents birth only (*N* = 1471).

The prevalence of late pregnancy PAE for WV residents only and according to the six predefined SAMSHA regions along with 95% CI are presented in Table 1. For WV residents only, the prevalence of late pregnancy PAE was 7.61% (95% CI: 6.26, 8.97) and the regional prevalence ranged from 2.27 to 17.11% (Fig. 1). Region 3 had one of the highest prevalence rates of late pregnancy PAE in the state. Table 2 provides the descriptive characteristics of the study population along with unadjusted OR and 95% CI of PAE and maternal and infant factors for the full dataset (*N* = 1,729). The descriptive characteristics of the study population by six SAMSHA regions are given in Table 3.

**Table 1:** State and SAMHSA regions frequencies, valid percentages, prevalence of late prenatal alcohol use (high PETH ≥8 ng/ml) and 95% confidence intervals.

SAMHSA region	*N*	Sample size by region (%)	Frequency for high PETH	Binomial proportion for high PETH (%)	95% lower confidence interval	95% upper confidence interval
**State**	**1471**	**—**	**112**	**7.61**	**6.26**	**8.97**
1	92	6.25	7	7.61	2.19	13.03
2	132	8.97	3	2.27	0.00	4.82
3	152	10.33	26	17.11	11.49	24.05
4	364	24.75	17	4.67	2.50	6.84
5	424	28.82	27	6.37	4.04	8.69
6	307	20.87	32	10.42	7.01	14.40

Region 1: Brooke, Hancock, Marshall, Ohio, Wetzel

Region 2: Berkeley, Grant, Hampshire, Hardy, Jefferson, Mineral, Morgan, Pendleton

Region 3: Calhoun, Jackson, Pleasants, Ritchie, Roane, Tyler, Wirt, Wood

Region 4: Barbour, Braxton, Doddridge, Gilmer, Harrison, Lewis, Marion, Monongalia, Preston, Randolph, Taylor, Tucker, Upshur

Region 5: Boone, Cabell, Clay, Kanawha, Lincoln, Logan, Mason, Mingo, Putnam, Wayne

Region 6: Fayette, Greenbrier, McDowell, Mercer, Monroe, Nicholas, Pocahontas, Raleigh, Summers, Webster, Wyoming

Bold: State average

**Table 2:** Descriptive characteristics and unadjusted odds ratios (OR) of late alcohol use during pregnancy and maternal and infant factors (*N* = 1729).

Perinatal factors	Total	Column %	Alcohol use during pregnancy				ORs (95% CI)
			No	Row %	Yes	Row %	
Birth weight (g)							
≤2000	47	2.72	39	82.98	8	17.02	**2.62 (1.19−5.79)**
2001−2500	111	6.42	101	90.99	10	9.01	1.27 (0.64−2.5)
2501−3000	357	20.65	323	90.48	34	9.52	1.35 (0.89−2.04)
>3000 (Ref.)	1214	70.21	1126	92.75	88	7.25	—
Health insurance							
Private insurance (Ref.)	786	49.94	724	92.11	62	7.89	—
Medicaid	788	50.06	723	91.75	65	8.25	1.05 (0.73–1.51)
Intrauterine substance use							
No (Ref.)	1476	85.37	1359	92.07	117	7.93	—
Yes	253	14.63	230	90.91	23	9.09	1.16 (0.73−1.86)
NAS							
No (Ref.)	1649	95.37	1517	92	132	8	—
Yes	80	4.63	72	10	8	10	1.28 (0.6−2.71)
NICU admission							
No (Ref.)	1613	93.29	1488	92.25	125	7.75	—
Yes	116	6.71	101	87.07	15	12.93	1.77 (0.99–3.13)
Feeding intention							
Bottle or both	756	43.72	683	90.34	73	9.66	**1.45 (1.02−2.04)**
Breast (Ref.)	973	56.28	906	93.11	67	6.89	—
Breastfed exclusively							
No	1109	64.14	1006	90.71	103	9.29	**1.61 (1.09−2.38)**
Yes (Ref.)	620	35.86	583	94.03	37	5.97	—
Gestational age (GA)							
Preterm	231	13.36	201	87.01	30	12.99	**1.88 (1.23−2.89)**
Term (Ref.)	1498	86.64	1388	92.66	110	7.34	—
Size for gestational age							
SGA	128	7.46	114	89.06	14	10.94	0.73 (0.36−1.48)
AGA (Ref.)	1440	83.92	1323	91.88	117	8.13	
LGA	148	8.62	139	93.92	9	6.08	1.39 (0.78−2.50)
Maternal age							
<19 (Ref.)	113	6.54	109	96.46	4	3.54	—
≥19	1616	93.46	1480	91.58	136	8.42	2.50 (0.91−6.89)
Maternal education							
≤10 grades	139	8.04	127	91.37	62	7.89	1.05 (0.73−1.51)
≥11 grades (Ref.)	1590	91.96	1462	91.75	65	8.25	—
Previous pregnancies							
0 (Ref.)	479	27.7	448	93.53	31	6.47	—
≥1	1250	72.3	1141	91.28	109	8.72	1.38 (0.91−2.09)
Smoking							
No (Ref.)	1356	78.43	1264	93.22	92	6.78	—
Yes	373	21.57	325	87.13	48	12.78	**2.03 (1.40−2.94)**
Race							
Other (Ref.)	134	7.88	124	92.54	10	7.46	—
White	1566	92.12	1440	91.95	126	8.05	1.09 (0.56−2.12)
Post-hoc analysis: Birth weight (g) in Term Births only (*n* = 1498)							
≤2000 and 2001–2500^a^	44	2.94	40	90.91	4	9.09	1.32 (0.46–3.77)
2501–3000	277	18.49	254	90.70	23	8.30	1.19 (0.74–1.93)
>3000 (Ref.)	1177	78.57	1094	92.95	83	7.05	—

Bold ORs = Statistically significant factors (*p* < 0.05)

*OR* odds ratio, *CI* confidence interval, *NAS* Neonatal Abstinence Syndrome

^a^Combining rows due to low cell count: ≤2000 frequency = 2 (0.13%) merged with 2001–2500 category frequency = 42 (2.8%)

**Table 3:** Descriptive characteristics of maternal factors at time of delivery in percentages (%) by SAMHSA regions (*N* = 1471).

SAMSHA regions	Age (≤19)	Race (White)	Education (≤10 grade)	Health insurance (Medicaid)	No. of previous pregnancy (none)	Alcohol use	Smoking	Substance use
**State**	**6.73%**	**91.29%**	**8.43%**	**54.25%**	**27.80%**	**7.61%**	**21.26%**	**15.30%**
1	5.43%	92.22%	3.26%	26.74%	25.00%	7.61%	28.26%	**21.74%**
2	6.82%	82.95%	9.85%	**63.08%**	26.52%	2.27%	17.42%	19.70%
3	7.89%	**97.30%**	9.87%	56.76%	31.58%	**17.11%**	21.05%	13.16%
4	4.95%	92.18%	8.79%	47.34%	**33.52%**	4.67%	18.41%	13.46%
5	6.84%	88.73%	6.60%	56.06%	25.00%	6.37%	18.87%	12.97%
6	**8.47%**	94.08%	**10.75%**	63.05%	24.43%	10.42%	**29.32%**	17.92%

Region 1: Brooke, Hancock, Marshall, Ohio, Wetzel

Region 2: Berkeley, Grant, Hampshire, Hardy, Jefferson, Mineral, Morgan, Pendleton

Region 3: Calhoun, Jackson, Pleasants, Ritchie, Roane, Tyler, Wirt, Wood

Region 4: Barbour, Braxton, Doddridge, Gilmer, Harrison, Lewis, Marion, Monongalia, Preston, Randolph, Taylor, Tucker, Upshur

Region 5: Boone, Cabell, Clay, Kanawha, Lincoln, Logan, Mason, Mingo, Putnam, Wayne

Region 6: Fayette, Greenbrier, McDowell, Mercer, Monroe, Nicholas, Pocahontas, Raleigh, Summers, Webster, Wyoming

Bold: State averages and the highest region for each factor

Next, we compared the prevalence of late pregnancy PAE established in this study (7.61%) against several other prevalence rates found using other methods, with the noninferiority research hypothesis that the prevalence would be “as good as or better than” the previously found prevalence rates, assuming these previous rates are underestimating the true prevalence. Results of the noninferiority testing demonstrate this testing method with a proportion of 7.61% to be “as good as or better than” the umbilical (*p*0 = 5.1%) and self-report testing (*p*0 = 2.7%) previously done, and “as good as or better than” the Texas state data utilizing the same method but with the >20 ng/ml cutoff (*p*0 = 8.4%), all *p* < 0.0001, within the 3% margin. However, noninferiority testing showed our prevalence rate was not “as good or better than” relative to the Texas state data using the equivalent LOQ cutoff of ≥ 8 ng/ml (*p*0 = 24.7%), *p* = 1.00.

The statistically significant factors associated with PAE included smoking during pregnancy, birth weight, gestational age, and breastfeeding intent and practice. The odds of smoking during pregnancy was twice in mothers with prenatal alcohol use vs. no use (OR: 2.03, 95% CI: 1.40, 2.94). The odds of having a preterm birth was also nearly twice among pregnant women with prenatal alcohol use vs. no use (OR: 1.88; 95% CI: 1.23, 2.89). Only one birth weight category was significantly associated with PAE [birth weight of ≤2000 g vs. >3000 g (OR: 2.62, 95%CI: 1.19, 5.79)]. The odds of not intending to exclusively breastfeed and not exclusively breastfeeding the newborn before hospital discharge was 2.62 (95% CI: 1.19, 5.79) and was 1.61 times (95% CI: 1.09, 2.38) among those with PAE vs. no exposure respectively.

The result of the post-hoc analysis for data restricted to full-term birth infants only showed that late PAE was not significantly associated with low birth weight (LBW) of <2500 g compared to >3000 g (Table 2). The second analysis for the association between PAE and preterm vs. term birth stratified by infant’s sex. In female infants the association was not statistically significant (OR: 1.85 95% CI: 0.95, 3.61, *p* = 0.0717) and in male infants this association was statistically significant (OR: 1.89 95% CI: 1.08, 3.31, *p* = 0.0269). The third post-hoc analysis showed that among all those infants with IUSE, 9.09% were positive for PAE. The IUSE did not capture 90.91% of the alcohol cases. Lastly, the prevalence of PAE in the month prior to birth using a higher cutoff of PETH > 20 ng/ml was 31 cases or 1.79% (95% CI: 1.17, 2.42) in the entire sample that included out-of-state residents as well.

## Discussion

Alcohol use during pregnancy is a significant public health issue globally, nationally, and also in the state of WV. This study used data from a sample of all residual DBS cards of infants born in the state from November 2017 to January 2018 to examine the prevalence of PAE using PETH. The results demonstrated that PAE in the last month prior to delivery was 8% among all births and 7.6% among WV residents. These prevalence rate of late pregnancy PAE is much higher than what was previously known for the state of WV.

The previous prevalence estimates for PAE for WV are from two sources. The first source is the PRAMS data from year 2015. These data are a self-reported telephone survey that asked women, (1) if they drank alcoholic drinks in the past 2 years; (2) if they drank alcohol 3 months before pregnancy; and (3) if they drank alcohol during the last 3 months of pregnancy. The result of the survey showed that 59.3% women drank in the past 2 years prior to pregnancy, 52.2% did not drink 3 months before pregnancy, and 2.7% (~1 in 40 women) drank during the last 3 months of pregnancy in WV.^9^ Data show that this method of data collection for alcohol use tends to underestimate the alcohol use prevalence by more than one third,^10^ perhaps due to social desirability bias, stigma, as well as fear of reports to Child Protective Services. The current study prevalence (7.61%) shows improvement in estimation over this previous self-report study (noninferiority *p* < 0.0001). The second source is a study conducted in eight diverse birthing hospitals in WV in 2009 using PETH in the umbilical cord tissue. The results from the umbilical cord tissue showed that on average 1 in 20 women (~5%) drank alcohol in late pregnancy.^18^ The current study showed the prevalence rates to be nearly 1 in 13 women (~8%), which is higher than what was previously reported and is statistically significant (noninferiority *p* < 0.0001). This may be due to the methodological differences in the study designs as well as in the data collection techniques and the type of sample used for the analysis.

We also compared our results to the recent statewide population base study conducted in Texas that used the same method as our study (i.e. detection of PETH in the infant residual DBS) and found the prevalence of PAE to be 24.7% when using a cutoff of ≥8 ng/ml and 8.4% when using a cutoff of >20 ng/ml.^13^ Using the ≥8 ng/ml PETH cutoff for comparison with the Texas study, we hypothesize that our study probably underestimated the prevalence rates of late pregnancy PAE in WV due to increased chance of false-negative results as a result of partial degradation of DBS sample that were analyzed 6−8 months after data collection. Moreover, there is no established PETH cutoff concentration that is used for assessing PAE in newborns.^27,28^ Some researchers have reported values above the limit of detection (LOD, ≥2 ng/ml) to indicate positive PAE,^27^ while others have proposed that future research should ascertain if PETH concentration in DBS samples between 2 and 8 ng/ml should be considered as light alcohol use and ≥8 ng/ml should be considered moderate to heavy alcohol use in pregnancy.^12^ For this study we used the cutoff of ≥8 ng/ml based on several previous reports suggesting the limit of quantification (LOQ) indicated any alcohol use.^12,14,16,22,23^ A previous validation article notes that this cutoff has 100% specificity and lower but comparable sensitivity to other methodologies to detect moderate chronic or intermittent binge drinking patterns during pregnancy.^27^ Although some researchers utilize a higher cutoff of ten times the LOD (>20 ng/ml) for identifying late pregnancy PAE,^13,28^ this higher cutoff was recommended for liquid blood specimens for the general population and not for the newborn population alone.^22^ In liquid blood samples the post-collection synthesis of PETH may occur if ethanol is present in the blood sample, resulting in false-positive results. However, no post-collection synthesis of PETH occurs in the DBS samples,^22,27^ and false-positive results are not possible without alcohol use; thus, it can be argued that any concentration above the LOD indicates alcohol use in late pregnancy.^12,29^ As there is no consensus on what the “ideal” cutoff is for newborns and no reports have identified false-positive PETH results without alcohol consumption, this study used a cutoff of ≥8 ng/ml (0.288 μmol/l) to identify any alcohol exposure 2–4 weeks prior to data collection.^30^

### Associated factors

The significant factors associated with PAE include smoking during pregnancy, LBW, gestational age, and breastfeeding. These findings are consistent with previous literature.^31–33^ Regarding preterm birth, the results showed that the odds of having a preterm birth among the prenatal alcohol use group nearly doubled compared to the no alcohol use group. For the birth weight variable, PAE (vs. no PAE) was significantly associated with LBW of ≤2000 g compared to the referent group of >3000 g. Two additional birth weight categories that include 2000–2500 g and 2501–3000 g were not statistically significantly associated with PAE compared to the referent group category of >3000 g. However, when the study was restricted to full-term birth infants only, the association between LBW of ≤2000 g compared to >3000 g became statistically not significant. Moreover, small for gestational age (SGA) vs. appropriate for gestational age (AGA) was also not a statistically significant factor associated with PAE. Previous studies have also shown mixed results regarding the association between PAE and preterm birth or SGA. Some studies have shown that there is no association between low to moderate PAE and SGA^34^ while others have shown there is an increased risk of SGA births in mothers who drink ≥3 units/day of alcohol in pregnancy.^35^ Our study is restricted to maternal alcohol use 2–4 weeks before delivery as well as having no data on the amount of alcohol consumption. Therefore, we were unable to assess or compare our findings to other studies that capture the amount of alcohol consumption at various time points throughout pregnancy.

### Regional prevalence

The six substate SAMSHA regional data for WV showed geographical disparities in the prevalence of late pregnancy PAE in WV. The mid-Ohio Valley Region 3 had one of the highest prevalence of late pregnancy PAE in the state. Our earlier work on IUSE in 2017 showed that Region 1, the northern panhandle region had one of the highest rates of IUSE (18.49%).^36^ In our current study, Region 3 ranked highest in PAE (17.11%) and fifth in IUSE (13.16%).

These disconcerting results led the research team to perform post-hoc analyses to determine if the linked IUSE data as reported by the statewide surveillance system (Project WATCH) at birth captured any, none, or all of the alcohol use (PETH) data. The results of the post-hoc analysis revealed that the substance use data did not capture more than 90% of the alcohol cases. The IUSE data in Project WATCH collects information from three sources, (1) self-report, (2) documented in the past medical records, or/and (3) positive drug screen. Self-reported alcohol use in pregnancy has low sensitivity and maternal blood, breath, or urine maybe only useful to detect recent alcohol exposure,^11^ demonstrating the challenges associated with timely identification and detection of PAE.

Further exploration into the demographic characteristics of the study population by regions showed that Region 3 consists of 97% White population, nearly 8% teenage pregnancies, and approximately 10% of the mothers had ≤10 grade of high school education. However, in the overall data for all births in the state, we observed that maternal race, age, and education were not statistically significantly associated with alcohol use in late pregnancy. Thus, at this point a follow-up study is required to understand the regional differences.

Limitations of the study include some of the reasons discussed earlier for differences in hypothesized prevalence rates: i.e., PETH is a direct alcohol biomarker, which indicates late pregnancy. PETH in the DBS is relatively stable over time if it is stored at lower temperatures.^15^ However, the DBS in our study were stored and shipped at room temperatures and analyzed for PETH after approximately 8 months, which likely resulted in a small amount of PETH degradation, consistent with Bakhireva et al.’s 2016 findings, which found temperature but not time minimally impacting PETH degradation over a 9-month period.^15^ Moreover, samples were collected in 3 months of November, December, and January and may have influenced the prevalence and the comparability to other samples collected over broad calendar periods, under the assumption that holiday drinking patterns could be higher than non-holidays drinking pattern. Additionally, the literature shows that there is no gold standard for detecting PAE and combining different screening tools and methods are needed to find the true prevalence rates in a population.^23^ Bracero et al. concluded that self-report, urine ethanol, and PETH testing combined identified more PAE cases than any single method.^23^ However, our study only used PETH in the infant residual DBS, and thus may have underestimated the prevalence rate. Finally, due to a lack of standardized PETH concentration cutoff, comparing the rates of PAE using DBS is difficult across studies. Although not the main purpose of this study, we were unable to assess the association for some of the main predictors of PAE such as pre-pregnancy alcohol consumption and exposure to abuse or violence^37^ due to lack of available data. Moreover, based on one peer-review comment, an additional post-hoc analysis was conducted to determine the association between PAE and preterm birth by infant’s sex. As further examining this association is beyond the scope of this study, we recommend future research to examine the association between PAE and preterm birth stratified by infant’s sex.

This study demonstrates that the rates of late pregnancy PAE in WV are high. Moreover, the detection of PETH in residual DBS was found to be an effective surveillance method to monitor rates of PAE for a broad epidemiological study.^22^ Comparisons against other methods showed improved prevalence estimates; however, using the LOQ for PETH likely still underestimates the true prevalence of any drinking during pregnancy. Analysis of the PETH data in conjunction with the statewide surveillance system (Project WATCH) identified many factors associated with PAE. Nearly 1 in 13 women in the state of WV drank alcohol in the last few weeks of pregnancy. Some substate regions experience as high as 1 in 6 women drinking alcohol in late pregnancy. The average number of births/year (2014−2018) for the state of WV is ~19,500 (SD = 1000.34), and an 8% PAE prevalence rate theoretically means that 1500 babies may have been exposed and at a risk of developing FASD. The prevalence estimates of late pregnancy PAE are alarming due to its association with the FASD and thus the findings of our study highlight the potential public health burden associated with PAE in WV as observed in four varying regions of the country as well.^6^

PAE is a serious public health problem. Developmental disabilities resulting from FASD are preventable and have high associated economic and societal costs.^38^ The effects of FASD may not always be apparent at birth; for conditions and complications developing later, such as at school age, it becomes increasingly likely that conditions due to FASD will be misdiagnosed or missed altogether.^39^ Knowing the true extent of the problem in a timely manner is the first step in identifying what resources and services are needed for primary, secondary, and tertiary prevention of this critical public health issue. Early detection of PAE and early diagnosis and intervention of FASD can prevent secondary disabilities and improve infant’s outcomes and the quality of life.^14,40^
